# Resveratrol, Metabolic Dysregulation, and Alzheimer’s Disease: Considerations for Neurogenerative Disease

**DOI:** 10.3390/ijms22094628

**Published:** 2021-04-28

**Authors:** Alex J. T. Yang, Ahmed Bagit, Rebecca E. K. MacPherson

**Affiliations:** 1 Department of Health Sciences, Brock University, St. Catharines, ON L2S 3A1, Canada; ay14dx@brocku.ca (A.J.T.Y.); ab15nf@brocku.ca (A.B.); 2 Centre for Neuroscience, Brock University, St. Catharines, ON L2S 3A1, Canada

**Keywords:** resveratrol, metabolic dysregulation, Alzheimer’s disease, neuroinflammation, SIRT1, AMPK, neuronal health, synaptic plasticity, PGC1-*α*

## Abstract

Alzheimer’s disease (AD) has traditionally been discussed as a disease where serious cognitive decline is a result of A*β*-plaque accumulation, tau tangle formation, and neurodegeneration. Recently, it has been shown that metabolic dysregulation observed with insulin resistance and type-2 diabetes actively contributes to the progression of AD. One of the pathologies linking metabolic disease to AD is the release of inflammatory cytokines that contribute to the development of brain neuroinflammation and mitochondrial dysfunction, ultimately resulting in amyloid-beta peptide production and accumulation. Improving these metabolic impairments has been shown to be effective at reducing AD progression and improving cognitive function. The polyphenol resveratrol (RSV) improves peripheral metabolic disorders and may provide similar benefits centrally in the brain. RSV reduces inflammatory cytokine release, improves mitochondrial energetic function, and improves A*β*-peptide clearance by activating SIRT1 and AMPK. RSV has also been linked to improved cognitive function; however, the mechanisms of action are less defined. However, there is evidence to suggest that chronic RSV-driven AMPK activation may be detrimental to synaptic function and growth, which would directly impact cognition. This review will discuss the benefits and adverse effects of RSV on the brain, highlighting the major signaling pathways and some of the gaps surrounding the use of RSV as a treatment for AD.

## 1. Introduction

In the last 20 years, the impact of chronic metabolic disease on neurogenerative diseases such as Alzheimer’s disease (AD) has become increasingly evident where metabolic or lifestyle factors (smoking, hypertension, physical inactivity, obesity, type-2-diabetes mellitus (T2D)) in combination represent 1/3 of reported AD cases [1]. With 26.8% of the Canadian population being obese, another 36.3% being overweight, and with a rapidly aging population (Stats Canada 2018), the push for an effective AD therapeutic has been more important than ever. It is for this reason that the polyphenol resveratrol (RSV, trans-3,4′,5-trihydroxystilbene) has gained so much attention as it has been reported to be effective at improving key pathways affected by metabolic diseases, such as inflammation and mitochondrial dysfunction [2,3]. This review will discuss the potential benefits of RSV on the brain, highlighting the major signaling pathways by which RSV is reported to exert neuroprotective effects, some of the gaps and potential adverse effects surrounding the use of RSV as a treatment for AD, as well as the important role RSV has played in progressing AD research.

## 2. Alzheimer’s Disease

AD impairs cognitive function by severely reducing brain volume. It is a neurodegenerative dementia that is typically described by its two main pathologies: amyloid-β (Aβ) plaques and neurofibrillary tangles (NFT) [4]. Both pathologies disrupt different aspects of neuron health and synaptic connectivity but may equally contribute to the cognitive decline observed with AD: Aβ-plaque development is driven by the oligomerization of large numbers of Aβ peptides physically damaging neural connections, whereas NFT destabilize microtubule connections, preventing the trafficking of protein/communication in neurons. While both Aβ and NFT are prevalent pathologies in AD, there is growing evidence for plaques and NFT being a late-stage result of other, underlying pathologies that precede the loss of brain matter [5]. Most notable is the research that describes the more sporadic form of AD and metabolic dysregulation.

### 2.1. Sporadic AD and Metabolic Dysregulation

Late-stage or sporadic AD (sAD) represents 95% of all reported AD cases [6]. Sporadic AD derives mostly from non-genetic factors; namely, environmental factors such as diet, physical inactivity, and various metabolic diseases [1,7,8]. Indeed, external risk factors including smoking, hypertension, depression, insulin resistance, diabetes, and obesity have been shown to account for ≈50% of all reported cases when combined [1]. Even improper sleep has been linked to increased risk of AD [9,10]. Many of these risk factors, such as T2D and obesity, contribute to what is described as metabolic dysregulation [11,12]. The commonality of underlying pathologies seen with these diseases such as low-grade chronic inflammation, hyperglycemia, hyperinsulinemia, hypertension, and impaired metabolic homeostasis are linked to the development of several metabolic pathologies such as insulin resistance and cardiovascular disease as well as the development of AD [13,14,15].

**Figure 1:**
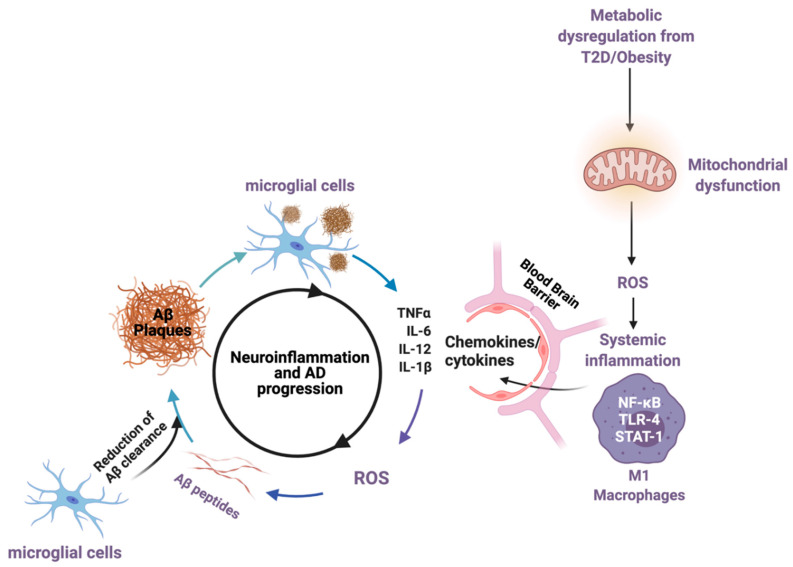
The impact of metabolic disease (T2D and obesity), mitochondrial dysregulation, and the generation of pro-inflammatory cytokines that cross the blood–brain barrier, inducing a cycle of neuroinflammation and increasing the rate of Aβ-plaque generation. Aβ peptides drive the development of ROS, mitochondrial dysfunction, and pro-inflammatory cytokines, which serves to increase AD pathology cyclically. Created by Biorender.com accessed on 30 March 2021.

While both Aβ plaques and NFT are considered hallmarks of sAD, there is an important role placed on the mechanisms that lead to the development of these hallmarks rather than the development themselves. These changes occur early in the disease progression—prior to cognitive symptoms—and involve increased inflammation and metabolic alterations that negatively impact neuronal health and, ultimately, cognitive function [16,17,18,19,20,21,22]. In fact, there is evidence to show that not only do increases in pro-inflammatory cytokines increase the Aβ plaque burden, but Aβ plaques will themselves induce increases in neuroinflammation and reactive oxygen species (ROS), promoting the development of NFT and worsening the associated cognitive decline in a vicious cycle (Figure 1) [17,23,24].

### 2.2. Type-2 Diabetes and Obesity

Our understanding of the impact that metabolic diseases such as T2D and obesity have on accelerating and contributing to AD pathologies has improved over the past 10 years [18,19]. Reductions in insulin-like-growth factor receptor (IGF-1Rβ) content, insulin degrading enzyme (IDE) content, and increased inhibitory insulin-receptor substrate phosphorylation (IRS1 S616) have all been reported in post-mortem AD brains, indicating that significant impairments in brain insulin signaling are identifiable in the disease [20,25,26,27,28]. Indeed, insulin resistance (IR) characteristic with T2D pathology has been linked to increased BACE1 activity [29,30], increased Aβ peptide content [31], and directly correlated to worse episodic, working, and global memory scores [20]. The development of brain IR has even been identified as an early step in the progression of AD [32]. Importantly, obesity has been linked to insulin resistance and T2D by inducing pro-inflammatory signaling cascades that inhibit insulin signaling [12,33,34]. Obesity is also a strong predictor of cognitive impairment, with neuroinflammation playing a key role in developing the observed reductions in brain volume [13,19,35,36,37,38]. This connection between peripheral factors including inflammation, hyperinsulinemia, and hyperglycemia and brain health serves to highlight the role that metabolic diseases play in AD and the promising impact that T2D therapeutics may have in the prevention and treatment of neurodegenerative diseases [7,19,39].

### 2.3. Neuroinflammation

(Neuro)inflammation offers an important connection for the metabolic impairments seen in AD [16,17]. The development of chronic, low-grade systemic inflammation is present early in both T2D and obesity, where pro-inflammatory cytokines are released from M1-type macrophages (TNF-α, IL-6, IL-12, IL-18) [36,37,40,41]. Under healthy conditions, these cytokines are secreted acutely to mitigate infection through increased ROS and nitrous oxide (NO) generation [42]. However, in pathological conditions characterized by low-grade chronic cytokine secretion, ROS generation is unregulated, causing major tissue damage, ER stress, and impaired insulin action in the liver, skeletal muscle, adipose, gut, and brain [11,43]. In the brain, these cytokines will induce an overactivation of microglial cells into a phenotype that limits Aβ peptide clearance, leading to increased Aβ-driven cytokine secretions and even glial-driven neuronal death [40,44]. This reduced Aβ clearance occurs alongside increased ROS generation, impaired cellular signaling, and mitochondrial impairments that have been brilliantly summarized here [21,22]. This type of glial cell activation likely occurs early in sAD progression [45], inducing a feedback loop for greater cytokine release with increased Aβ processing, and it can lead to further impairments in brain insulin signaling [46].

### 2.4. Metabolic Dysregulation and Synaptic Plasticity

The impact of metabolic dysregulation on synaptic strength and signaling cannot be ignored when discussing AD. The cognitive decline observed in AD can be related to reduced expression of genes directly related to synapse health/function [47] and a significant loss of the dendritic spine density of neural networks at early stages of the disease [48]. Synapses will strengthen/prune themselves in response to increases/decreases of stimulation (i.e., synaptic firing events), and a loss of synaptic networking directly impacts cognitive function [47,49,50]. While substantial research has examined how Aβ impairs synaptic transmission rates in later stages of AD [51,52,53], there remains a significant role for alterations in protein expression and synapse translocation in modifying the strength of synaptic connections, particularly at early time points in the progression of the disease [54,55]. The chronic metabolic stress, observed with neuroinflammation and IR, has been shown to impair aspects of synaptic transmission, such as the regulation synaptic protein expression/translation, transmission rates, and even the growth of new synapses [20,50,56,57,58,59,60,61,62]. These highlight significant factors that likely impact early sAD development and merit therapeutic discussion.

### 2.5. Resveratrol and Alzheimer’s Disease

Resveratrol (RSV, 3,5,4′-trihydroxystilbene) is a naturally occurring polyphenol typically found in the skin of grapes, red wine, rhubarb, and several other plants [63]. RSV has gained interest in health research due to its therapeutic potential for metabolic diseases, such as insulin resistance and T2D [64]. Furthermore, RSV has been described to have some neuroprotective effects related to cognitive decline [65]. For example, Moussa et al. [66] demonstrated that 52 weeks of RSV treatment, in patients with mild to moderate AD (1 g RSV daily), prevented the age-related decline that was observed in the control group in a mini-mental state exam. Similarly, a study in overweight older adults (50–80 years, BMI = 25–30 kg/m^2^) showed that 26 weeks of oral RSV treatment (200 mg/d) lead to increases in memory performance, memory retention, and functional hippocampal connectivity (1000 Functional Connectomes project) [67]. These studies highlight RSV as a promising therapeutic for neurodegenerative diseases. As a polyphenol, these beneficial effects of RSV have been linked to its role as an antioxidant and anti-inflammatory agent.

The beneficial effects of RSV in the periphery or in relation to whole body metabolism have been well characterized. For example, an early study demonstrated that supplementing mice with RSV (22.4 mg/kg/day) for 6 months alongside a high-calorie diet resulted in improved bodyweight and insulin/glucose sensitivity compared to mice fed a high-calorie diet without RSV supplementation [68]. This was also accompanied by increases in mitochondrial content and reductions in fat accumulation in the liver [68]. These aforementioned results indicate that RSV has a substantial effect on preventing major pathologies that are known to induce an inflammatory response. Moreover, to investigate RSV’s anti-inflammatory activity in the periphery, Cui et al. incorporated an average dietary supplement of 11.93 mg/kg/day of RSV to 8–12 weeks old C57BL/6 mice and demonstrated that RSV significantly reduces the content of known inflammation indicators, such as the inducible nitric oxide synthase iNOS and necrosis tumor factor-α (TNF-α) in the colon [69,70]. These anti-inflammatory effects have also been mirrored in studies that utilized AD-diagnosed human participants. For example, Moussa et al. showed that CSF levels of IL-4 were found to be elevated following RSV treatment (1 g/day 52 weeks) and that the RSV administration prevented an increase in TNF-α content seen within the control group, which is indicative of RSV-driven anti-inflammatory action [66]. Additionally, the results also showed that the RSV group had a significant reduction of IL-8 in plasma, which is a pro-inflammatory cytokine implicated in attracting and activating neutrophils during inflammatory responses, [66,71]. Ongoing clinical trials show that resveratrol shows promise in improving glycemic control and reducing insulin resistance in type 2 diabetic and obese patients potentially through anti-inflammatory mechanisms [72,73]. However, a recent meta-analysis of clinical trials of RSV has also indicated that more research is required for adequate conclusions to be drawn surrounding RSV’s effectiveness for T2D [74]. These results, together, point to RSV playing a role in the regulation of inflammation, as well as the induction of adaptive immunity which, points to the therapeutic potential of RSV in a disease such as AD [66].

To confirm that the effects of RSV extend to the brain, Broderick et al. [75] has shown that RSV supplementation in a transgenic AD mouse model (3 × Tg-AD mice; 4 g/kg/day for 5 months) had a marked reduction in pro-inflammatory markers (NF-κB, PARP, GFAP) that were seen with controls. As well, Zhang et al. [76] also demonstrated that RSV prevents LPS-mediated microglial activation, as well as the release of the pro-inflammatory markers, TNF-α, nitric oxide, and IL-1β in rat primary cortical neuron–glia cultures. Ma et al. [77] also demonstrated that IL-1β and IL-6 hippocampal and pre-frontal cortex content were markedly reduced with RSV supplementation (25 mg/kg/d for 5 weeks) in a concurrent model of T2D and AD (Wistar rats, streptozotocin IP and Aβ hippocampal injections, respectively) which also showed improvements in cognitive function. It is important to note that both Zhang et al. [76] and Ma et al. [77] only saw these reductions when cells were challenged (LPS or AD model) before RSV was administered; RSV alone saw no differences in cytokine release compared to controls, suggesting that a metabolic challenge is required in order to see RSV-driven benefits. It is difficult to ascribe the protective effects on cognitive function as being a consequence of RSV-induced reductions in inflammation; however, given the role of neuroinflammation in inducing the progression of AD, RSV’s ability to modulate neuroinflammation as well as improvements in cognition have certainly made RSV a therapeutically promising compound.

One of the known contributing factors to neuroinflammatory signaling is the unregulated production of reactive oxygen species (ROS). ROS are highly volatile radical oxygen species that are a product of aerobic respiration. ROS expression is important for acute cellular signaling; however, its overproduction will induce major cellular damage and increase inflammatory cytokine release in response [78]. ROS is also implicated in AD, as the aggregation of amyloid-beta peptides has been shown to induce microglia activation, which in turn increases pro-inflammatory cytokine release, subsequently increasing ROS production (Figure 1) [79]. The high metabolic demand of astrocytes and neurons makes them highly susceptible to the mitochondrial dysfunction common in AD, which causes a greater production of ROS [80]. Importantly, a part of RSV’s anti-inflammatory action has been shown to reduce ROS production in both the periphery and the brain. Song et al. demonstrated that RSV (50 µM/L) could significantly reduce ROS production in peripheral cells by reducing iNOS content as well as upregulating the activity of the ROS scavenger superoxide dismutase (SOD) in a human cell model (human aortic endothelial cells) [81]. Similarly, Huang et al. show that RSV (100 mM/5 µL) injected in the brains of adult male Sprague–Dawley rats, exposed to Aβ, showed a marked reduction in iNOS content and an increase in a free radical scavenger heme oxygenase-1, indicating that RSV’s anti-oxidative action can take place in the brain [82]. Ma et al. [77] also showed in their model of T2D and AD that RSV (25 mg/kg/d for 5 weeks) significantly upregulated the activity of antioxidant enzymes superoxide dismutase (SOD) and glutathione (GSH) in both the hippocampus and prefrontal cortex. This evidence points to the ability of RSV to modulate ROS content and demonstrates its potential in improving AD pathology. However, while promising, the precise mechanisms by which RSV exerts these actions were not addressed in these studies and require further discussion.

### 2.6. Resveratrol and SIRT1

In AD pathogenesis, one of the pathways that Aβ peptides acts on to induce neuronal death is the activation of apoptotic proteins by ROS. These proteins, such as p53, can induce apoptosis through the activation of mitogen-activated protein kinase, JNK [79]. However, it has been shown that the NAD-dependent deacetylating enzyme sirtuin 1 (SIRT1) prevents the activation and content of p53, reduces neurodegeneration via apoptosis, and even prevents pro-inflammatory cytokine release through increased SOD regulation of ROS [83,84,85,86,87]. Importantly, RSV has been shown to act through SIRT1 to exert these protective properties. An investigation by Kim et al. showed that following the administration of RSV (5 µg/µL, ICV injection, 2–3×/week for 3 weeks) in AD transgenic mice (p25), p53 content was significantly reduced within the hippocampus and neurodegenerative neuron loss was prevented, indicating that RSV can increase neuronal survival [84]. A growing body of literature also suggests that SIRT1 plays a major role in facilitating RSV’s anti-inflammatory and anti-oxidative activity through interacting with downstream target proteins [85]: Zou et al. [86] showed that RSV-mediated SIRT1 activity reduced the content of pro-inflammatory cytokines IL-1β and IL-18 and upregulated the antioxidant content of SOD and GSH (traumatic brain injury Sprague–Dawley rats. 100 mg/kg; IP injection); Ma et al. [77] also prescribed their increases in SOD and GSH activity as being by a RSV–SIRT1 driven action.

**Figure 2:**
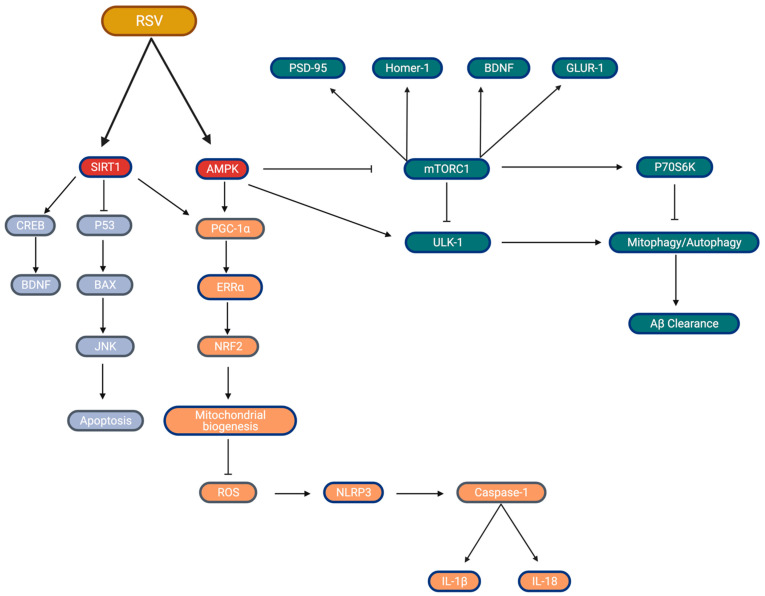
Major markers of the RSV signaling cascade proposed to be beneficial toward brain health. (Blue) SIRT1 inhibition of p53 leads to a reduction in apoptosis, increases in cellular BDNF content; (Orange) SIRT1/AMPK upregulation of PCG1-α expression and activity lead to improvements in biogenesis, preventing ROS accumulation, subsequently leading to reductions in inflammatory markers; (Green) AMPK increases in autophagy-driven Aβ-peptide clearance through mTORC1 inhibition, potential impacts on synaptic plasticity and cognition. Created by Biorender.com accessed on 30 March 2021.

Recent studies have demonstrated that SIRT1 likely facilitates RSV’s action in the context of metabolic regulation [88,89]. Current research has established a strong relationship between metabolic dysregulation and the deterioration in mitochondrial health [90]. More importantly, mitochondrial dysfunction has been associated with neurodegeneration and AD through the increased dependence on oxidative phosphorylation due to aging [78]. The increase in oxidative phosphorylation, coupled with the reduction in aerobic glycolysis, results in increases of ROS production, which further drives metabolic dysregulation [78]. On the other hand, research has shown that RSV can improve mitochondrial health and protect against metabolic dysregulation [91]. One such mechanism, where RSV action can enhance mitochondrial health, is through increasing the expression and activation of PGC1-α, which is a major inductor of mitochondrial biogenesis [91]. Mitochondrial biogenesis has been shown to reduce the production of superoxide radicals through increasing the activity of complexes III, which in turn protects against oxidative stress [92]. Furthermore, another factor essential in the activation of PGC-1α is AMP-activated protein kinase (AMPK). AMPK is a known major regulator of metabolism and plays a significant role in modulating energy homeostasis and when activated is involved in inducing the transcription of several proteins important to metabolic regulation, including PGC-1α [93]. It has also been shown that SIRT1-mediated increases in mitochondrial biogenesis require AMPK: Price et al. showed that mice overexpressing SIRT1 (no RSV) demonstrated marked increases in skeletal muscle AMPK activity that were completely absent in SIRT1 KO mice (6 months old C57BL/6J), even when treated with resveratrol (25 mg/kg/day and 215 mg/kg/day for 8 months). Additionally, in the absence of SIRT1, RSV’s administration did not yield significant increases of mtDNA, PGC1-α content, or mitochondrial respiration, all of which were seen in the SIRT1 KI mice [93]. Therefore, while Price et al. [93] specifically examined skeletal muscle, this evidence indicates that SIRT1 (both alone and activated by RSV)-mediated improvements in mitochondrial health and function require AMPK, which may translate to the brain, and that SIRT1 is a major target by which RSV may exert its neuroprotective properties (Figure 2).

### 2.7. Resveratrol and AMPK

Alongside the importance of the SIRT1/AMPK interaction, RSV has also been shown to activate AMPK directly to exert a key set of metabolic benefits: improving mitochondrial content/function (increased PGC1-α content, increased activity of COXIV, and citrate synthase), improvements in insulin sensitivity and glucose uptake (increased GLUT4 translocation, reduced IGF-I, improved Akt signaling), increased autophagic/mitophagic clearance of cell debris, and increased FFA mobilization (increased PDH, PDK4, ATGL content) [94,95,96,97,98,99]. These metabolic alterations are well documented to occur in several peripheral tissues including skeletal muscle, liver, heart, and adipose tissue and have all been directly related to improvements in T2D and obesity pathology [97,98,99,100,101]. Activation by RSV occurs directly through AMPKs upstream kinases, LKB1 and CAMKKβ, but also indirectly by modifying the AMP/ATP ratio through impairing ATP production by the inhibition of mitochondrial complex V (F_1_F_0_-ATPase/ATP synthase) [68,102]. The direct regulation of these metabolic pathways has been what has made AMPK a prime candidate for T2D and obesity, particularly within the liver and skeletal muscle [103,104,105,106,107]. However, AMPK has garnered specific attention in the brain as an AD therapeutic for the improvements in mitochondrial function and neuroinflammation, but also for the reductions in Aβ content that are seen through AMPK-mediated autophagy [85,103,104,105,106,107].

RSV has been confirmed, in vitro, to induce a number of AMPK-dependent findings in the brain such as increases in PGC1-α and TFAM content, and COXIV activity [108,109,110,111], as well as reductions in oxidative stress and pro-inflammatory signaling (increased SOD activity, reduced iNOS, ROS, Nf-κB), mirroring some major results shown in peripheral tissues [104]. Along those same lines, Ma et al. [77] showed in a diabetic AD model (8–10 mo Wistar rats; diabetes conferred via streptozotocin IP) that orally ingested RSV daily (25 mg/kg; 5 weeks) is capable of inducing SIRT1 activation and lowering Il-6 content in both the hippocampus and pre-frontal cortex. Orsu et al. [112] confirmed similar results where a 20 mg/kg RSV treatment in a model of cerebral ischemia and reperfusion (adult Wistar rats; RSV injected 5 min before reperfusion via IP) was sufficient to reduce TNF-α and IL-6 brain content. Results demonstrating the RSV-mediated reductions in pro-inflammatory cytokines in the brain have been further confirmed to occur in multiple animal models [113,114] and even in circulation for some human studies [66]. Particularly in a study by Qi et al. [115], direct intracerebroventricular RSV injection (0.2 mg/kg/day for 10 days) resulted in significant reductions in NF-κB, IL-1β, and NLRP3 pro-inflammatory signalling in an AD mouse model (Kunming mice; AD induced by intracerebral injection of Aβ). These observed reductions in pro-inflammatory signaling coincided with increases in AMPK activity and SIRT1 expression. Importantly, Qi et al. [115] confirmed that intracerebroventricular injection of RSV prevented cognitive impairments, seen with Aβ-plaque development. Qi et al. demonstrated improved dynamic spatial working memory (Y-maze test) and improved escape latency (Morris Water Maze), showing that the direct administration of RSV will prevent AD-driven pathological impairments. Similarly, Sarroca et al. [116] also recently demonstrated some neuroprotective benefits of RSV by showing that an orally fed dose of RSV (120 mg/kg/day; 16 weeks) was protective against a number of AD pathologies (increased APP content, BACE1 activity) as well as prevented impairments in cognitive behavior (open field test, novel object recognition test) in a high-fat mouse model of AD (C57BL/6, 60% kcal from fat).

Indeed, there is evidence for RSV being protective against AD pathologies particular through AMPK (such as altering Aβ content) in both metabolic and genetic models of AD. Vingtdeux et al. [117] showed that RSV (400 mg/kg) was detectable in APP/PS1 transgenic mouse brains following oral ingestion and that RSV induced an upregulation in AMPK-dependent autophagy, leading to an improved clearance of Aβ peptides and an overall reduction in plaque size. This activation of RSV/AMPK-driven autophagy was also confirmed by our lab in a high-fat diet induced mouse model (100 mg/kg RSV, 60% kcal from fat) [107] as well as in an ischemic mouse model, where RSV (1.8 mg/kg) was delivered via intravenous tail injection [94]. Results from all these studies confirm that not only is RSV capable of entering the brain but also that activation of AMPK with RSV has the potential to improve overall AD pathology, likely through improved ROS management, reduced mitochondrial dysfunction, reduced inflammation, and increased autophagic clearance of Aβ peptides [103,110,118,119].

It must be mentioned that RSV-driven AMPK activation has been reported to have varying effects based on cell type (adipocyte, hepatocyte, myocyte, etc.) [96]. As well, most research, showing RSV-meditated improvements/prevention on metabolic syndrome through SIRT1 or AMPK, has been thoroughly demonstrated in peripheral tissues rather than the brain [68,93,96,99,102,106,120,121]. As such, the evidence supporting the role of RSV in mitigating metabolic syndrome in the brain and regulating AD pathology is promising, particularly in the case of Qi et al. [115]. However, given that the majority of studies in vivo examined RSV within models that will affect other tissues alongside the brain, it becomes difficult to determine if major improvements in cognitive function/AD pathologies are related to the direct effect of RSV on the brain or if they arise from whole-body improvements given the multi-tissue promiscuity of RSV.

### 2.8. Resveratrol, Synaptic Plasticity, and Cognitive Function

Much of this review has focused on the molecular signaling of RSV and the impact that its supplementation has on well-known and discussed risk factors of metabolic impairment (neuroinflammation, insulin resistance, mitochondrial function). However, while RSV shows promise as a therapeutic, through improvements in mitochondrial biogenesis/efficiencies and reductions in pro-inflammatory cytokines/ROS content (Figure 2), with respect to AD, it is important to discuss the impact of RSV on synaptic plasticity and cognitive function. Given the broad nature of what cognitive function implies (memory formation, object recognition, critical thinking, etc.), for the purposes of this review, the main focus will be on the impact that RSV has on synaptic function, which is more often described as synaptic plasticity as it pertains to the cognitive changes that have been reported.

Synaptic plasticity describes the ability of a synapse connection to increase (long-term potentiation; LTP) or decrease (long-term depression; LTD) synaptic transmission rates. These changes in synaptic strength require changes in the coordination and expression of proteins at both sides the synapse. Some of the major proteins commonly discussed are BDNF, cyclic-AMP response element-binding protein (CREB), Post-synaptic density markers PSD-95 and Homer-1, as well as markers of neurotransmitter release synaptobrevin, synaptophysin, and SNAP-25 [55,122,123]. Increased coordination of these proteins is linked to increasing LTP and has been directly linked to improved memory and cognitive function [47,124,125], and the loss of these proteins has been observed in models of metabolic dysregulation and aging where major cognitive deficiencies are reported [126]. Given the role of SIRT1 on inducing CREB and BDNF expression [85], as well as the role of AMPK on early synaptic gene expression [58,59], the discussions surrounding RSV improving cognitive function are not without merit.

In support of this, several studies have shown that treatment of mice with RSV does result in improvements in memory and cognitive function: Labban et al. [127] saw improvements in a passive avoidance task in an AD mouse model with RSV (male SWR/J mice, 40 mg/kg/day RSV via IP, AlCl_3_ induced NFT), Palomera-Avalos et al. [128] showed improvements in novel object recognition (NORT) and open field tests (OFT) in a metabolically impaired model (22 month old C57BL/6J mice, high-fat diet (HFD) 60% calories from fat, 160 mg/kg/day RSV from chow), and Cao et al. [129] (Male/female Sprague–Dawley rats, 100 mg/kg/day RSV from chow) similarly showed RSV-induced cognitive improvements in *t*-test exploration/success rates as well as improvements in Morris Water Maze (MWM) escape latency. RSV has also been reported to induce increases in early-LTP markers Arc and PSA-NCAM, BDNF [130], and late-LTP markers synaptophysin, PSD95, PSD93, and CREB in models of cerebral vascular disease [131], as well as regulate AMPAR expression and NDMA-mediated Ca^2+^ influx [132]. These studies are promising in that they suggest that RSV can improve global cognition in disease states and that at least a portion of RSV’s improvements could come from improved synaptic protein expression, which is likely driven through SIRT1 activation of CREB [133].

Many studies report that RSV induces pro-synaptic plasticity effects, be it through changes in LTP-inducing proteins or morphological changes in synaptic structure [127,129,130,132,133]. While it is certainly true that increased synaptic expression of LTP-inducing proteins [47] and morphological changes [60] can be directly linked to improvements in cognition, it is important to demonstrate that these changes in expression will result in the increased translocation of key proteins to the synapse, and that this results in improvements in synaptic transmission rates. This is most evident in how plasticity has been discussed in previous, well-written reviews on RSV and hippocampal plasticity [134]. In fact, very few studies present electrophysiological data to show RSV directly improving synaptic transmission rates in either culture or brain. Wang et al. [135] demonstrated through whole-cell patch-clamp recordings of cultured hippocampal neurons that 4 h of RSV treatment (40 µM) resulted in a marked increase in LTP induction. This change in LTP amplitude was also demonstrated to occur with upregulation of AMPAR specific-receptor expression but not NMDAR, which was also confirmed in vivo (C57BL/6 male mice, 30 mg/kg RSV via IP, age not specified). These results were promising as they did connect increases in a major synaptic protein (AMPAR) to an increase in LTP conductance. A drawback of this study was that it was conducted on cultured neurons and did not address the impact of RSV on LTP in a model of metabolic stress [135]. Li et al. [131], did demonstrate that mice treated with RSV (40 mg/kg RSV via IP) showed increased LTP response via field excitatory postsynaptic potential (fEPSP) of the dentate gyrus hippocampal region that protected against LTP impairments of chronic cerebral hypoperfusion. Tong et al. [136] showed similar results in mouse hippocampal brain slices (C57BL/6 mice) that RSV (100 µM) was able to facilitate an increase in LTP (via fEPSP) and that RSV could rescue impairments in LTP in the presence of α-synuclein. Tong et al. confirmed the findings of Wang et al. in which RSV seems to promote the increased synaptic expression of AMPARs. It is important to note that RSV was not administered in vivo via diet or IP injection in this study, but rather RSV was delivered ex vivo in the brain bath solution and allowed to continually perfuse (10 min) during fEPSP recordings, which limits the physiological relevance of these findings. Overall, these three studies present compelling data that RSV can directly regulate synaptic function to promote LTP. However, none of the three aforementioned studies directly examined the impact of RSV treatment on LTP development in a model of metabolic dysregulation which, given the impact of metabolically-driven pathologies on the progression of AD, certainly merits discussion. As well, Hsieh et al. [132] presented data in which RSV treatment in cultured hippocampal cells did not result in an increase in LTP potential at an equivalent dose and method to Tong et al. (100 µM). Additionally, Wang et al. reported a reduction in PSD-95 synaptic content that was AMPK-dependent [135].

In fact, there is research that suggests that RSV may be detrimental to neurogenesis and further evidence that AMPK activation is detrimental toward synaptic plasticity and health. Similar to the studies presented above, Park et al. [111] treated C57BL/6 male mice as well as neural-progenitor cells (NPC) with RSV (10 mg/kg via IP; or 20 µM for cell culture) for 14 days to examine the role of RSV on NPC proliferation in situ and neuronal cell proliferation/neurogenesis in vivo. In contrast to several studies we have previously mentioned, Park et al. showed that both in vitro and in vivo RSV significantly impaired NPC proliferation, further impaired the differentiation of newly generated neurons of the hippocampus, and reduced the expression of both BDNF and CREB. This was shown to occur at least partially through an AMPK-mediated action, as treatment with the AMPK inhibitor, compound c (CC; 5 µM), ablated NPC proliferation reductions seen with RSV, and thus, RSV treatment was confirmed to impact spatial learning/memory (MWM) [111]. It is important to note that this was demonstrated in young mice (4 weeks), during a period of time in which the mouse brain is still developing [137], indicating that RSV may only be detrimental during development. However, with evidence that neurogenesis still occurs in adult brains [138], the impact that RSV has on synaptic plasticity and brain health merits more investigation, particularly in models of aging and metabolic dysregulation.

An interesting finding from Park et al. was that AMPK played a partial role in mediating RSV impairments. Despite the benefits that AMPK activation has been shown to confer in T2D and obesity and even in spite of some of the evidence presented in this review, there is counterintuitive evidence showing AMPK hyperactivity in both post-mortem AD brains (Braak stage 6) [56,139] as well as in HFD mouse brains [30,128,140]. These findings question the benefits of RSV on regulating brain health under conditions of metabolic dysregulation, particularly through AMPK. Indeed, there is evidence that demonstrates that chronic AMPK activity might, in fact, be detrimental to neuronal health, as cultured hippocampal neurons treated with the AMPK activator 5-aminoimidazole-4-carboxamide-1-β-d-ribofuranoside (AICAR, 2 µM) and metformin (200 µM) significantly reduce axon length, total neuron growth, and neuronal polarization [60].

There is also evidence to show that AMPK plays a major role in both early brain development as well as inducing early-LTP synaptic protein gene expression (Arc, c-Fos, Egrl) in mature brains [58]. As well, mTORC1, the major cellular proliferation complex that is downstream of AMPK, requires a balanced activation in order to induce increases in major proteins associated with synaptic strength (PSD-95, GluR1, Synapsin), indicating that AMPK activation would likely be detrimental to neuronal health [141]. Indeed, the extent of the AMPK-mTORC1 interaction has been demonstrated to directly impact LTP strength in the brain. Potter et al. [50] demonstrated in 4–6-week-old C57BL/6 mice that the activation of AMPK by both AICAR (1 mM) and metformin (5 µM) resulted in significant impairments in late-LTP development: impairments which were restored with AMPK inhibition by AMPK inhibitor Compound C (CC, 1 µM). Potter et al. further demonstrated that the restorations in late-LTP, via AMPK inhibition, were abolished with the potent mTORC1 inhibitor, rapamycin (1 µM), confirming that the restorations in late-LTP are mTORC1 driven [50]. Ma et al. [56] similarly examined the impact of AMPK inhibition on LTP development in a genetic mouse model of AD (10–12 m old APP/PS1 mice), where hippocampal slices treated with CC (5 µM) showed significant improvements in LTP. This restoration was also paired with increased de novo protein synthesis, which is impaired in APP/PS1 and directly linked to synaptic impairments [56]. However, it is important to note that these studies examining the impact of AMPK activation on LTP were conducted with drugs administered during fEPSP testing and have yet to be demonstrated to impair global cognitive function in tests such as the MWM.

Taken together, the hyperactivation of AMPK is detrimental, and the interaction between AMPK and mTORC1 directly impacts synaptic function. However, it is likely that the regulated, timely, activation of AMPK is required for proper neuronal health [58,141]. This corresponds with the idea that the brain becomes less capable of handling stressful events, such as increased pro-inflammatory cytokine release, insulin resistance, or increase FFA accumulation as individuals age [21]. To highlight this idea of a balanced activation of pathways such as SIRT1 or AMPK, Palomera et al. [142] showed that aged mice (24 month) did not respond to 160 mg/kg RSV following an inflammatory lipopolysaccharide challenge (saw no reductions in TLR4, IL-6, or TNF-α), lending credence to the idea that mismanagement of cellular stressors will impair the cellular ability to deal with metabolic stressors such as T2D, obesity, or neuroinflammation. This impaired state of metabolism brought on by age is likely exacerbated, as Aβ peptides accumulate in AD and likely initiate a cycle of further impairments in neuroinflammation, making individuals more suspectable to developing metabolic syndrome [22]. It is for this reason one could consider a metabolic disease such as AD as the acceleration of normal brain aging [47] and may possibly define the differences in disease progression between the early-onset development with FAD (driven mainly by increases in plaque/tangles by genetic factors) and the late-onset, slower development seen with sAD. In fact, this idea might be what explains some of the variations in the RSV LTP results observed by Park et al. [111], as the authors had no metabolic or AD-like pathology challenge in their model (IR, HFD, APP mutant, aged mice, etc.).

### 2.9. Resveratrol Bioavalibiltiy and Toxicity

The papers referenced in this review cite a wide range of RSV dosages (1.8 mg/kg to 1000 mg/day), differing methods of delivery (Oral, IP, ICV), as well as variations in the models that were utilized (cell culture, mouse, rat, and human). This makes it difficult to translate these findings toward a relevant physiological dose in humans. If RSV is going to be considered for preventative therapy of AD, it becomes imperative to establish a range at which RSV is most effective. Importantly, previous work has demonstrated that high dosages of RSV (3 g/kg in rats) induce pro-oxidative effects, including DNA damage, apoptosis, and nephrotoxicity [143,144,145]. These high-dose side effects become particularly relevant when discussing the effectiveness of RSV to reach the brain, as the pharmacokinetics of RSV are quite poor: low bioavailability and low solubility greatly affect the amount of RSV that circulates unmetabolized (oral dose of 25 mg = <5.0 ng/mL in plasma) [146]. However, RSV is indeed capable of crossing the BBB (0.0456 mg/kg; lumbar puncture and external carotid, confirmed by brain tissue HPLC analysis) [147] and, with the exception of Vingtdeux et al. [117] who used a dose of 400 mg/kg/day in mice, the majority of oral, animal doses lie between 100 and 200 mg/kd/day, which are well tolerated by animals with no side effects and show significant changes in brain signaling. It should be noted that the high doses of RSV that were shown to result in apoptosis and nephrotoxicity (3 g/kg/d for 4 weeks) in rats translate to a very high human equivalent dose of ≈33.87 g/d (weighing 70 kg) based on equivalent dose calculations for humans [148] and that a 0.3 g/kg/d dosage (≈3.3 g/d in humans weighing 70 kg) was also tested and showed no kidney or liver toxicity [145]. Overall, while several studies examining the effects of RSV in the brain utilize animal models, the doses used translate to well-tolerated doses in humans. Most human trials also report that RSV is well tolerated at up to 5 g/day with mild side effects (diarrhea, nausea, headaches), indicating that a large dose of RSV is required to elicit toxicity [143,144,145,149]. For a more detailed discussion surrounding RSV tolerance and the dosage reported in both human and animal models, please refer to the reviews by Rahman et al. [150] and Ramirez et al. [3].

While there is ample human testing that remains to be done, the few human studies that have tested RSV on AD and/or cognitive function have shown conflicting results: two class II clinical trials on mild to moderate AD patients have been conducted over long periods (52 weeks) that showed no reduction in CSF Aβ content following RSV treatments (500 mg/d up to 1 g/d and 1 g twice daily) [66,151]. As well, while RSV was not confirmed to cause any reductions in cognitive function as no cognitive tests were conducted, Turner et al. [152] showed a significant reduction in brain volume (MRI) of participants treated with RSV for 52 weeks. Two other studies by Wightman et al. [151] and Kennedy et al. [153] also showed that treatment with resveratrol (250 mg and 250 mg or 500 mg respectably) did not affect cognitive function. However, these results were shown in healthy individuals with a single dose of RSV where measurements including cognitive testing were taken after a 45-min absorption period [151,153].

## 3. Conclusions

Presently, our understanding of AD mainly revolves around the physical loss of brain matter that directly impairs cognition due to Aβ-plaque accumulation and NFT development. In the brain, RSV shows promise for the treatment of AD by improving cognitive function and reducing AD pathologies such as Aβ. These benefits seem to be driven by improvements in brain metabolism through the regulation of ROS production, inflammation, and by preventing mitochondrial dysfunction. With this review, we have highlighted that the benefits of RSV on brain health address a more significant issue: that the detrimental changes in brain metabolism drive the severity of cognitive decline. The impact of inflammation and mitochondrial dysfunction on neuronal health and AD pathology alone speaks to the impact that metabolic disease has on cognition and shows the promise of RSV as an intervention. Overall, the evidence presented in this review shows that the use of RSV is promising for the treating this underlying pathology of AD. As well, the use of RSV in AD has begun to shift the narrative toward AD being a metabolically susceptible disease. However, there are conflicting results toward the benefits of RSV on cognition that remain to be fully explored, particularly regarding RSV’s regulation of synaptic health and function. This becomes even more evident when considering the impact that one of the major downstream targets of RSV, AMPK, has on regulating protein synthesis and on synaptic plasticity. Future work should aim to determine exactly how/if RSV changes cognitive function.

## Data Availability

Not applicable.

## Abbreviations

AD	Alzheimer’s Disease
RSV	Resveratrol
Aβ	Amyloid-β
NFT	Neurofibrillary Tangles
sAD	Sporadic Alzheimer’s Disease
FAD	Familial Alzheimer’s Disease
ROS	Reactive Oxygen Species
T2D	Type-2-Diabetes
IGF-1Rβ	Insulin-Like-Growth Factor Receptor
IDE	Insulin-Degrading-Enzyme
NO	Nitrous Oxide
SIRT1	Sirtuin 1
AMPK	Amp-Kinase
SOD	Superoxide Dismutase
LTP	Long-Term Potentiation
LTD	Long-Term Depression
CREB	Cyclic-AMP Response Element-Binding Protein
BDNF	Brain-Derived Neurotrophic Factor
NORT	Novel-Object Recognition Test
OFT	Open Field Test
MWM	Morris Water Maze
fEPSP	Field Excitatory Postsynaptic Potential
NPC	Neural-Progenitor Cells
CC	Compound C
AICAR	5-Aminoimidazole-4-Carboxamide-1-Β-D-Ribofuranoside
mTORC1	Mammalian Target Of Rapamycin Complex 1
HFD	High-Fat Diet
TNF-α	Tumor Necrosis Factor α
